# Strong Convergence Algorithm for Split Equilibrium Problems and Hierarchical Fixed Point Problems

**DOI:** 10.1155/2014/390956

**Published:** 2014-02-20

**Authors:** Abdellah Bnouhachem

**Affiliations:** ^1^School of Management Science and Engineering, Nanjing University, Nanjing 210093, China; ^2^Ibn Zohr University, ENSA, BP 1136, Agadir, Morocco

## Abstract

The purpose of this paper is to investigate the problem of finding the approximate element of the common set of solutions of a split equilibrium problem and a hierarchical fixed point problem in a real Hilbert space. We establish the strong convergence of the proposed method under some mild conditions. Several special cases are also discussed. Our main result extends and improves some well-known results in the literature.

## 1. Introduction

Let *H* be a real Hilbert space, whose inner product and norm are denoted by 〈·, ·〉 and ||·||. Let *C* be a nonempty closed convex subset of *H*. We introduce the following definitions which are useful in the following analysis.

**Definition 1**

The mapping *T*:  *C* → *H* is said to be

(a) monotone, if
  (1) $$\begin{array}{c} \langle Tx-Ty,x-y\rangle \geq 0,\;\forall x,y\in C; \end{array}$$
(b) strongly monotone, if there exists *α* > 0 such that
  (2) $$\begin{array}{c} \langle Tx-Ty,x-y\rangle \geq \alpha {||x-y||}^{2},\;\forall x,y\in C; \end{array}$$
(c) *α*-inverse strongly monotone, if there exists *α* > 0 such that
  (3) $$\begin{array}{c} \langle Tx-Ty,x-y\rangle \geq \alpha {||Tx-Ty||}^{2},\;\forall x,y\in C; \end{array}$$
(d) nonexpansive, if
  (4) $$\begin{array}{c} ||Tx-Ty||\leq ||x-y||,\;\forall x,y\in C; \end{array}$$
(e) *k*-Lipschitz continuous, if there exists a constant *k* > 0 such that
  (5) $$\begin{array}{c} ||Tx-Ty||\leq k||x-y||,\;\forall x,y\in C; \end{array}$$
(f) contraction on *C*, if there exists a constant 0 ≤ *k* < 1 such that
  (6) $$\begin{array}{c} ||Tx-Ty||\leq k||x-y||,\;\forall x,y\in C. \end{array}$$

It is easy to observe that every *α*-inverse strongly monotone *T* is monotone and Lipschitz continuous. It is well known that every nonexpansive operator *T*:  *H* → *H* satisfies, for all (*x*, *y*) ∈ *H* × *H*, the inequality

(7) 〈(x−T(x))−(y−T(y)),T(y)−T(x)〉  ≤12||(T(x)−x)−(T(y)−y)||2

and therefore, we get, for all (*x*, *y*) ∈ *H* × Fix⁡(*T*),

(8) $$\begin{array}{c} \langle x-T\left(x\right),y-T\left(x\right)\rangle \leq \frac{1}{2}{||T\left(x\right)-x||}^{2}. \end{array}$$

See, for example, [1, Theorem 1], and [2, Theorem 3].

The fixed point problem for the mapping *T* is to find *x* ∈ *C* such that

(9) $$\begin{array}{c} Tx=x. \end{array}$$

We denote by *F*(*T*) the set of solutions of (9). It is well known that *F*(*T*) is closed and convex and *P*_*F*_(*T*) is well defined (see [3]).

The equilibrium problem denoted by EP is to find *x* ∈ *C* such that

(10) $$\begin{array}{c} F\left(x,y\right)\geq 0,\;\forall y\in C. \end{array}$$

The solution set of (10) is denoted by *EP*⁡(*F*). Numerous problems in physics, optimization, and economics reduce to finding a solution of (10); see [4–7]. In 1997, Combettes and Hirstoaga [8] introduced an iterative scheme of finding the best approximation to the initial data when *EP*⁡(*F*) is nonempty. In 2007, Plubtieng and Punpaeng [6] introduced an iterative method for finding the common element of the set *F*(*T*)∩*EP*⁡(*F*).

Recently, Censor et al. [9] introduced a new variational inequality problem which we call the split variational inequality problem (SVIP). Let *H*_1_ and *H*_2_ be two real Hilbert spaces. Given operators *f*:  *H*_1_ → *H*_1_ and *g*:  *H*_2_ → *H*_2_, a bounded linear operator *A*:  *H*_1_ → *H*_2_, and nonempty, closed, and convex subsets *C*⊆*H*_1_ and *Q*⊆*H*_2_, the SVIP is formulated as follows: find a point x* ∈ *C* such that

(11) $$\begin{array}{c} \langle f\left(x^{*}\right),x-x^{*}\rangle \geq 0\;\forall x\in C \end{array}$$

and such that

(12) y∗=Ax∗∈Q  solves  〈g(y∗),y−y∗〉≥0                 ∀y∈Q.

In [10], Moudafi introduced an iterative method which can be regarded as an extension of the method given by Censor et al. [9] for the following split monotone variational inclusions:

(13) $$\begin{array}{c} \text{Find}\;x^{*}\in H_{1}\;\;\text{such}\;\text{that}\;0\in f\left(x^{*}\right)+B_{1}\left(x^{*}\right) \end{array}$$

and such that

(14) $$\begin{array}{c} y^{*}=Ax^{*}\in H_{2}\;\;\text{solves}\;0\in g\left(y^{*}\right)+B_{2}\left(y^{*}\right), \end{array}$$

where *B*_*i*_: *H*_*i*_ → 2^*H*_*i*_^ is a set-valued mapping for *i* = 1,2. Later Byrne et al. [11] generalized and extended the work of Censor et al. [9] and Moudafi [10].

Very recently, Kazmi and Rizvi [12] studied the following pair of equilibrium problems called split equilibrium problem: let *F*_1_:  *C* × *C* → *R* and *F*_2_: *Q* × *Q* → *R* be nonlinear bifunctions and let *A*: *H*_1_ → *H*_2_ be a bounded linear operator; then, the split equilibrium problem (SEP) is to find x* ∈ *C* such that

(15) $$\begin{array}{c} F_{1}\left(x^{*},x\right)\geq 0,\;\forall x\in C, \end{array}$$

and such that

(16) $$\begin{array}{c} y^{*}=Ax^{*}\in Q\;\;\text{solves}\;F_{2}\left(y^{*},y\right)\geq 0,\;\forall y\in Q. \end{array}$$

The solution set of SEP (15)-(16) is denoted by Λ = {*p* ∈ *EP*⁡(*F*_1_) : *Ap* ∈ *EP*⁡(*F*_2_)}.

Let *S*: *C* → *H* be a nonexpansive mapping. The following problem is called a hierarchical fixed point problem: find *x* ∈ *F*(*T*) such that

(17) $$\begin{array}{c} \langle x-Sx,y-x\rangle \geq 0,\;\forall y\in F\left(T\right). \end{array}$$

It is known that the hierarchical fixed point problem (17) links with some monotone variational inequalities and convex programming problems; see [13, 14]. Various methods [15–20] have been proposed to solve the hierarchical fixed point problem. In 2010, Yao et al. [14] introduced the following strong convergence iterative algorithm to solve the problem (17):

(18) $$\begin{array}{c} y_{n}=\beta_{n}Sx_{n}+\left(1-\beta_{n}\right)x_{n},\\ x_{n+1}=P_{C}\left[\alpha_{n}f\left(x_{n}\right)+\left(1-\alpha_{n}\right)Ty_{n}\right],\;\forall n\geq 0, \end{array}$$

where *f*:  *C* → *H* is a contraction mapping and {*α*_*n*_} and {*β*_*n*_} are two sequences in (0,1). Under some certain restrictions on parameters, Yao et al. proved that the sequence {*x*_*n*_} generated by (18) converges strongly to *z* ∈ *F*(*T*), which is the unique solution of the following variational inequality:

(19) $$\begin{array}{c} \langle \left(I-f\right)z,y-z\rangle \geq 0,\;\forall y\in F\left(T\right). \end{array}$$

In 2011, Ceng et al. [21] investigated the following iterative method:

(20) $$\begin{array}{c} x_{n+1}=P_{C}\left[\alpha_{n}\rho U\left(x_{n}\right)+\left(I-\alpha_{n}\mu F\right)\left(T\left(y_{n}\right)\right)\right],\;\forall n\geq 0, \end{array}$$

where *U* is a Lipschitzian mapping and *F* is a Lipschitzian and strongly monotone mapping. They proved that under some approximate assumptions on the operators and parameters, the sequence {*x*_*n*_} generated by (20) converges strongly to the unique solution of the variational inequality

(21) $$\begin{array}{c} \langle \rho U\left(z\right)-\mu F\left(z\right),x-z\rangle \geq 0,\;\forall x\in \;\mathrm{Fix}\left(T\right). \end{array}$$

In the present paper, inspired by the above cited works and by the recent works going in this direction, we give an iterative method for finding the approximate element of the common set of solutions of (15)-(16) and (17) in real Hilbert space. Strong convergence of the iterative algorithm is obtained in the framework of Hilbert space. We would like to mention that our proposed method is quite general and flexible and includes many known results for solving split equilibrium problems and hierarchical fixed point problems; see, for example, [13, 14, 17–19, 21–23] and relevant references cited therein.

## 2. Preliminaries

In this section, we recall some basic definitions and properties, which will be frequently used in our later analysis. Some useful results proved already in the literature are also summarized. The first lemma provides some basic properties of projection onto *C*.

**Lemma 2**

Let *P*_*C*_ denote the projection of *H* onto *C*. Then, one has the following inequalities:

(22)  〈z−PC[z],PC[z]−v〉≥0, ∀z∈H,  v∈C;〈u−v,PC[u]−PC[v]〉≥||PC[u]−PC[v]||2, ∀u,v∈H; ||PC[u]−PC[v]||≤||u−v||, ∀u,v∈H;||u−PC[z]||2≤||z−u||2−||z−PC[z]||2, ∀z∈H,  u∈C.

**Assumption 3 (see [24])**

Let *F*:  *C* × *C* → ℝ be a bifunction satisfying the following assumptions:

(i) *F*(*x*, *x*) = 0, for all *x* ∈ *C*;
(ii) *F* is monotone; that is, *F*(*x*, *y*) + *F*(*y*, *x*) ≤ 0, for all *x*, *y* ∈ *C*;
(iii) for each *x*, *y*, *z* ∈ *C*, lim⁡_*t*→0_ *F*(*tz* + (1 − *t*)*x*, *y*) ≤ *F*(*x*, *y*);
(iv) for each *x* ∈ *C*, *y* → *F*(*x*, *y*) is convex and lower semicontinuous;
(v) for fixed *r* > 0 and *z* ∈ *C*, there exists a bounded subset *K* of *H*_1_ and *x* ∈ *C*∩*K* such that
  (23) $$\begin{array}{c} F\left(y,x\right)+\frac{1}{r}\langle y-x,x-z\rangle \geq 0,\;\forall y\in C\setminus K. \end{array}$$

**Lemma 4 (see [8])**

Assume that *F*_1_: *C* × *C* → ℝ satisfies Assumption 3. For *r* > 0 and for all *x* ∈ *H*_1_, define a mapping *T*_*r*_^*F*_1_^: *H*_1_ → *C* as follows:

(24) TrF1(x)={z∈C: F1(z,y)+1r〈y−z,z−x〉≥0,  ∀y∈C}.

Then the following hold:

(i) *T*_*r*_^*F*_1_^ is nonempty and single-valued;
(ii) *T*_*r*_^*F*_1_^ is firmly nonexpansive; that is,
  (25) ||TrF1(x)−TrF1(y)||2≤〈TrF1(x)−TrF1(y),x−y〉,                   ∀x,y∈H1;
(iii) *F*(*T*_*r*_^*F*_1_^) = *EP*(*F*_1_);
(iv) *EP*(*F*_1_) is closed and convex.

Assume that *F*_2_:  *Q* × *Q* → ℝ satisfies Assumption 3. For *s* > 0 and for all *u* ∈ *H*_2_, define a mapping *T*_*s*_^*F*_2_^:  *H*_2_ → *Q* as follows:

(26) TsF2(u)={v∈Q:F2(v,w)+1s〈w−v,v−u〉  ≥0,∀w∈Q}.

Then *T*_*s*_^*F*_2_^ satisfies conditions (i)–(iv) of Lemma 4. Consider *F*(*T*_*s*_^*F*_2_^) = *EP*⁡(*F*_2_, *Q*), where *EP*⁡(*F*_2_, *Q*) is the solution set of the following equilibrium problem:

(27) $$\begin{array}{c} \text{find}\;y^{*}\in Q\;\;\text{such}\;\text{that}\;F_{2}\left(y^{*},y\right)\geq 0,\;\forall y\in Q. \end{array}$$

**Lemma 5 (see [25])**

Assume that *F*_1_:  *C* × *C* → ℝ satisfies Assumption 3, and let *T*_*r*_^*F*_1_^ be defined as in Lemma 4. Let *x*, *y* ∈ *H*_1_ and *r*_1_, *r*_2_ > 0. Then

(28) $$\begin{array}{c} ||T_{r_{2}}^{F_{1}}\left(y\right)-T_{r_{1}}^{F_{1}}\left(x\right)||\leq ||y-x||+\left|\frac{r_{2}-r_{1}}{r_{2}}\right|||T_{r_{2}}^{F_{1}}\left(y\right)-y||. \end{array}$$

**Lemma 6 (see [26])**

Let *C* be a nonempty closed convex subset of a real Hilbert space *H*. If  *T*:  *C* → *C* is a nonexpansive mapping with Fix⁡(*T*) ≠ *∅*, then the mapping *I* − *T* is demiclosed at 0; that is, if {*x*_*n*_} is a sequence in *C* weakly converging to *x* and if {(*I* − *T*)*x*_*n*_} converges strongly to 0, then (*I* − *T*)*x* = 0.

**Lemma 7 (see [21])**

Let *U*:  *C* → *H* be *τ*-Lipschitzian mapping and let *F*:  *C* → *H* be a *k*-Lipschitzian and *η*-strongly monotone mapping; then for 0 ≤ *ρτ* < *μη*, *μF* − *ρU* is *μη* − *ρτ*-strongly monotone; that is,

(29) 〈(μF−ρU)x−(μF−ρU)y,x−y〉≥(μη−ρτ)||x−y||2,                      ∀x,y∈C.

**Lemma 8 (see [27])**

Suppose that *λ* ∈ (0,1) and *μ* > 0. Let *F*:  *C* → *H* be an *k*-Lipschitzian and *η*-strongly monotone operator. In association with nonexpansive mapping *T*:  *C* → *C*, define the mapping *T* ^*λ*^:  *C* → *H* by

(30) $$\begin{array}{c} T^{\lambda }x=Tx-\lambda \mu FT\left(x\right),\;\forall x\in C. \end{array}$$

Then *T* ^*λ*^ is a contraction provided that *μ* < (2*η*/*k* ^2^); that is,

(31) $$\begin{array}{c} ||T^{\lambda }x-T^{\lambda }y||\leq \left(1-\lambda \nu \right)||x-y||,\;\forall x,y\in C, \end{array}$$

where $\nu =1-\sqrt{1-\mu (2\eta -\mu L^{2})}$.

**Lemma 9 (see [28])**

Assume that {*a*_*n*_} is a sequence of nonnegative real numbers such that

(32) $$\begin{array}{c} a_{n+1}\leq \left(1-\gamma_{n}\right)a_{n}+\delta_{n}, \end{array}$$

where {*γ*_*n*_} is a sequence in (0,1) and *δ*_*n*_ is a sequence such that

1. ∑_*n*=1_^*∞*^ *γ*_*n*_ = *∞*;
2. limsup⁡_*n*→*∞*_ *δ*_*n*_/*γ*_*n*_ ≤ 0 or ∑_*n*=1_^*∞*^|*δ*_*n*_| < *∞*.

Then lim⁡_*n*→*∞*_ *a*_*n*_ = 0.

**Lemma 10 (see [29])**

Let *C* be a closed convex subset of *H*. Let {*x*_*n*_} be a bounded sequence in *H*. Assume that

- the weak *w*-limit set *w*_*w*_(*x*_*n*_) ⊂ *C* where *w*_*w*_(*x*_*n*_) = {*x*:  *x*_*n*_*i*__⇀*x*};
- for each *z* ∈ *C*, lim⁡_*n*→*∞*_||*x*_*n*_ − *z*|| exists.

Then {*x*_*n*_} is weakly convergent to a point in *C*.

## 3. The Proposed Method and Some Properties

In this section, we suggest and analyze our method and we prove a strong convergence theorem for finding the common solutions of the split equilibrium problem (15)-(16) and the hierarchical fixed point problem (17).

Let *H*_1_ and *H*_2_ be two real Hilbert spaces and let *C*⊆*H*_1_ and *Q*⊆*H*_2_ be nonempty closed convex subsets of Hilbert spaces *H*_1_ and *H*_2_, respectively. Let *A*:  *H*_1_ → *H*_2_ be a bounded linear operator. Assume that *F*_1_: *C* × *C* → ℝ and *F*_2_: *Q* × *Q* → ℝ are the bifunctions satisfying Assumption 3 and *F*_2_ is upper semicontinuous in first argument. Let *S*, *T*:  *C* → *C* be a nonexpansive mapping such that Λ∩*F*(*T*) ≠ *∅*. Let *F*:  *C* → *C* be an *k*-Lipschitzian mapping and *η*-strongly monotone and let *U*:  *C* → *C* be *τ*-Lipschitzian mapping. Now we introduce the proposed method as follows.

**Algorithm 11**

For a given *x*_0_ ∈ *C* arbitrarily, let the iterative sequences {*u*_*n*_}, {*x*_*n*_}, and {*y*_*n*_} be generated by

(33) $$\begin{array}{c} u_{n}=T_{r_{n}}^{F_{1}}\left(x_{n}+\gamma A^{*}\left(T_{r_{n}}^{F_{2}}-I\right)Ax_{n}\right);\\ y_{n}=\beta_{n}Sx_{n}+\left(1-\beta_{n}\right)u_{n};\\ x_{n+1}=P_{C}\left[\alpha_{n}\rho U\left(x_{n}\right)+\left(I-\alpha_{n}\mu F\right)\left(T\left(y_{n}\right)\right)\right],\;\forall n\geq 0, \end{array}$$

where {*r*_*n*_}⊂(0,2*ς*) and *γ* ∈ (0,1/*L*), *L* is the spectral radius of the operator A*A, and A* is the adjoint of *A*. Suppose that the parameters satisfy 0 < *μ* < (2*η*/*k* ^2^), 0 ≤ *ρτ* < *ν*, where $\nu =1-\sqrt{1-\mu (2\eta -\mu k^{2})}$. And {*α*_*n*_} and {*β*_*n*_} are sequences in (0,1) satisfying the following conditions:

- lim⁡_*n*→*∞*_ *α*_*n*_ = 0 and ∑_*n*=1_^*∞*^ *α*_*n*_ = *∞*;
- lim⁡_*n*→*∞*_(*β*_*n*_/*α*_*n*_) = 0;
- ∑_*n*=1_^*∞*^ | *α*_*n*−1_ − *α*_*n*_ | <*∞* and ∑_*n*=1_^*∞*^|*β*_*n*−1_ − *β*_*n*_| < *∞*;
- liminf⁡_*n*→*∞*_ *r*_*n*_ < limsup⁡_*n*→*∞*_ *r*_*n*_ < 2*ς* and ∑_*n*=1_^*∞*^|*r*_*n*−1_ − *r*_*n*_| < *∞*.

**Remark 12**

Our method can be viewed as extension and improvement for some well-known results as follows

- The proposed method is an extension and improvement of the method of Wang and Xu [23] for finding the approximate element of the common set of solutions of a split equilibrium problem and a hierarchical fixed point problem in a real Hilbert space.
- If the Lipschitzian mapping *U* = *f*, *F* = *I*, *ρ* = *μ* = 1, we obtain an extension and improvement of the method of Yao et al. [14] for finding the approximate element of the common set of solutions of a split equilibrium problem and a hierarchical fixed point problem in a real Hilbert space.
- The contractive mapping *f* with a coefficient *α* ∈ [0,1) in other papers (see [14, 19, 22, 27]) is extended to the cases of the Lipschitzian mapping *U* with a coefficient constant *γ* ∈ [0, *∞*).

This shows that Algorithm 11 is quite general and unifying.

**Lemma 13**

Let x* ∈ Λ∩*F*(*T*). Then {*x*_*n*_},  {*u*_*n*_}, and {*y*_*n*_} are bounded.

**Proof**

Let x* ∈ Λ∩*F*(*T*); we have x* = *T*_*r*_*n*__^*F*_1_^(x*) and Ax* = *T*_*r*_*n*__^*F*_2_^(Ax*). Then

(34) ||un−x∗||2=||TrnF1(xn+γA∗(TrnF2−I)Axn)−x∗||2=||TrnF1(xn+γA∗(TrnF2−I)Axn)−TrnF1(x∗)||2≤||xn+γA∗(TrnF2−I)Axn−x∗||2=||xn−x∗||2+γ2||A∗(TrnF2−I)Axn||2 +2γ〈xn−x∗,A∗(TrnF2−I)Axn〉=||xn−x∗||2 +γ2〈(TrnF2−I)Axn,AA∗(TrnF2−I)Axn〉 +2γ〈xn−x∗,A∗(TrnF2−I)Axn〉.

From the definition of *L*, it follows that

(35) γ2〈(TrnF2−I)Axn,AA∗(TrnF2−I)Axn〉  ≤Lγ2〈(TrnF2−I)Axn,(TrnF2−I)Axn〉  =Lγ2||(TrnF2−I)Axn||2.

It follows from (8) that

(36) 2γ〈xn−x∗,A∗(TrnF2−I)Axn〉  =2γ〈A(xn−x∗),(TrnF2−I)Axn〉  =2γ〈A(xn−x∗)+(TrnF2−I)Axn     −(TrnF2−I)Axn,(TrnF2−I)Axn〉  =2γ(〈TrnF2Axn−Ax∗,(TrnF2−I)Axn〉     −||(TrnF2−I)Axn||2)  ≤2γ(12||(TrnF2−I)Axn||2−||(TrnF2−I)Axn||2)  =−γ||(TrnF2−I)Axn||2.

Applying (36) and (35) to (34) and from the definition of *γ*, we get

(37) ||un−x∗||2  ≤||xn−x∗||2+γ(Lγ−1)||(TrnF2−I)Axn||2  ≤||xn−x∗||2.

Denote *V*_*n*_ = *α*_*n*_ *ρU*(*x*_*n*_)+(*I* − *α*_*n*_ *μF*)(*T*(*y*_*n*_)). Next, we prove that the sequence {*x*_*n*_} is bounded; without loss of generality we can assume that *β*_*n*_ ≤ *α*_*n*_ for all *n* ≥ 1. From (33), we have

(38) ||xn+1−x∗||  =||PC[Vn]−PC[x∗]||  ≤||αnρU(xn)+(I−αnμF)(T(yn))−x∗||  ≤αn||ρU(xn)−μF(x∗)||   +||(I−αnμF)(T(yn))−(I−αnμF)T(x∗)||  =αn||ρU(xn)−ρU(x∗)+(ρU−μF)(x∗)||   +||(I−αnμF)(T(yn))−(I−αnμF)T(x∗)||  ≤αnρτ||xn−x∗||+αn||(ρU−μF)(x∗)||   +(1−αnν)||yn−x∗||  ≤αnρτ||xn−x∗||+αn||(ρU−μF)(x∗)||   +(1−αnν)||βnSxn+(1−βn)un−x∗||  ≤αnρτ||xn−x∗||+αn||(ρU−μF)(x∗)||   +(1−αnν)(βn||Sxn−Sx∗||+βn||Sx∗−x∗||         +(1−βn)||un−x∗||)  ≤αnρτ||xn−x∗||+αn||(ρU−μF)(x∗)||   +(1−αnν)(βn||Sxn−Sx∗||+βn||Sx∗−x∗||         +(1−βn)||xn−x∗||)  ≤αnρτ||xn−x∗||+αn||(ρU−μF)(x∗)||   +(1−αnν)(βn||xn−x∗||+βn||Sx∗−x∗||         +(1−βn)||xn−x∗||)  =(1−αn(ν−ρτ))||xn−x∗||   +αn||(ρU−μF)(x∗)||   +(1−αnν)βn||Sx∗−x∗||  ≤(1−αn(ν−ρτ))||xn−x∗||   +αn||(ρU−μF)(x∗)||+βn||Sx∗−x∗||  ≤(1−αn(ν−ρτ))||xn−x∗||   +αn(||(ρU−μF)(x∗)||+||Sx∗−x∗||)  =(1−αn(ν−ρτ))||xn−x∗||   +αn(ν−ρτ)ν−ρτ(||(ρU−μF)x∗||+||Sx∗−x∗||)  ≤  max⁡{||xn−x∗||,1ν−ρτ      ×(||(ρU−μF)(x∗)||+||Sx∗−x∗||)},

where the third inequality follows from Lemma 8.

By induction on *n*, we obtain ||*x*_*n*_ − x*|| ≤ max⁡{||*x*_0_ − x*||, (1/(1 − *ρ*))(||(*ρU* − *μF*)x*|| + ||Sx* − x*||)}, for *n* ≥ 0 and *x*_0_ ∈ *C*. Hence {*x*_*n*_} is bounded and, consequently, we deduce that {*u*_*n*_}, {*y*_*n*_}, {*S*(*x*_*n*_)}, {*T*(*x*_*n*_)}, {*F*(*T*(*y*_*n*_))}, and {*U*(*x*_*n*_)} are bounded.

**Lemma 14**

Let x* ∈ Λ∩*F*(*T*) and {*x*_*n*_} the sequence generated by the Algorithm 11. Then one has

- lim⁡_*n*→*∞*_||*x*_*n*+1_ − *x*_*n*_|| = 0;
- the weak *w*-limit set *w*_*w*_(*x*_*n*_) ⊂ *F*(*T*), (*w*_*w*_(*x*_*n*_) = {*x* : *x*_*n*_*i*__⇀*x*}).

**Proof**

Since *u*_*n*_ = *T*_*r*_*n*__^*F*_1_^(*x*_*n*_ + γA*(*T*_*r*_*n*__^*F*_2_^ − *I*)*Ax*_*n*_) and *u*_*n*−1_ = *T*_*r*_*n*−1__^*F*_1_^(*x*_*n*−1_ + γA*(*T*_*r*_*n*−1__^*F*_2_^ − *I*)*Ax*_*n*−1_)  it follows from Lemma 5 that

(39) ||un−un−1||  ≤||xn−xn−1    +γ(A∗(TrnF2−I)Axn−A∗(Trn−1F2−I)Axn−1)||   +|1−rn−1rn|||TrnF1(xn+γA∗(TrnF2−I)Axn)         −(xn+γA∗(TrnF2−I)Axn)||  ≤||xn−xn−1−γA∗A(xn−xn−1)||   +γ||A||||TrnF2Axn−Trn−1F2Axn−1||   +|1−rn−1rn|||TrnF1(xn+γA∗(TrnF2−I)Axn)         −(xn+γA∗(TrnF2−I)Axn)||  ≤(||xn−xn−1||2−2γ||A(xn−xn−1)||2    +γ2||A||4||xn−xn−1||2)1/2   +γ||A||(||A(xn−xn−1)||       +|1−rn−1rn|||TrnF2Axn−Axn||)   +|1−rn−1rn|||TrnF1(xn+γA∗(TrnF2−I)Axn)         −(xn+γA∗(TrnF2−I)Axn)||  ≤(1−2γ||A||2+γ2||A||4)1/2||xn−xn−1||   +γ||A||2||xn−xn−1||   +γ||A|||1−rn−1rn|||TrnF2Axn−Axn||   +|1−rn−1rn|||TrnF1(xn+γA∗(TrnF2−I)Axn)         −(xn+γA∗(TrnF2−I)Axn)||  =(1−γ||A||2)||xn−xn−1||   +γ||A||2||xn−xn−1||   +γ||A|||1−rn−1rn|||TrnF2Axn−Axn||   +|1−rn−1rn|||TrnF1(xn+γA∗(TrnF2−I)Axn)         −(xn+γA∗(TrnF2−I)Axn)||  =||xn−xn−1||+γ||A|||1−rn−1rn|||TrnF2Axn−Axn||   +|1−rn−1rn|||TrnF1(xn+γA∗(TrnF2−I)Axn)         −(xn+γA∗(TrnF2−I)Axn)||  =||xn−xn−1||+|rn−rn−1rn|(γ||A||σn+χn),

where *σ*_*n*_ : = ||*T*_*r*_*n*__^*F*_2_^ *Ax*_*n*_ − *Ax*_*n*_|| and *χ*_*n*_ : = ||*T*_*r*_*n*__^*F*_1_^(*x*_*n*_ + γA*(*T*_*r*_*n*__^*F*_2_^ − *I*)*Ax*_*n*_) − (*x*_*n*_ + γA*(*T*_*r*_*n*__^*F*_2_^ − *I*)*Ax*_*n*_)||. Without loss of generality, let us assume that there exists a real number *μ* such that *r*_*n*_ > *μ* > 0, for all positive integers *n*. Then we get

(40) $$\begin{array}{c} ||u_{n-1}-u_{n}||\leq ||x_{n-1}-x_{n}||+\frac{1}{\mu }\left|r_{n-1}-r_{n}\right|\left(\gamma ||A||\sigma_{n}+\chi_{n}\right). \end{array}$$

From (33) and the above inequality, we get

(41) ||yn−yn−1||  =||βnSxn+(1−βn)un    −(βn−1Sxn−1+(1−βn−1)un−1)||  =||βn(Sxn−Sxn−1)+(βn−βn−1)Sxn−1    +(1−βn)(un−un−1)+(βn−1−βn)un−1||  ≤βn||xn−xn−1||+(1−βn)||un−un−1||   +|βn−βn−1|(||Sxn−1||+||un−1||)  ≤βn||xn−xn−1||+(1−βn)   ×{||xn−1−xn||+1μ|rn−1−rn|(γ||A||σn+χn)}   +|βn−βn−1|(||Sxn−1||+||un−1||)  ≤||xn−xn−1||+1μ|rn−1−rn|  (γ||A||σn+χn)   +|βn−βn−1|(||Sxn−1||+||un−1||).

Next, we estimate

(42) ||xn+1−xn|| =||PC[Vn]−PC[Vn−1]|| ≤||αnρ(U(xn)−U(xn−1))+(αn−αn−1)ρU(xn−1)   +(I−αnμF)(T(yn))−(I−αnμF)T(yn−1)   +(I−αnμF)(T(yn−1))−(I−αn−1μF)(T(yn−1))|| ≤αnρτ||xn−xn−1||+(1−αnν)||yn−yn−1||  +|αn−αn−1|(||ρU(xn−1)||+||μF(T(yn−1))||),

where the second inequality follows from Lemma 8. From (41) and (42), we have

(43) ||xn+1−xn||  ≤αnρτ||xn−xn−1||+(1−αnν)   ×{||xn−xn−1||+1μ|rn−1−rn|(γ||A||σn+χn)     +|βn−βn−1|(||Sxn−1||+||un−1||)}   +|αn−αn−1|(||ρU(xn−1)||+||μF(T(yn−1))||)  ≤(1−(ν−ρτ)αn)||xn−xn−1||   +1μ|rn−1−rn|(γ||A||σn+χn)   +|βn−βn−1|(||Sxn−1||+||un−1||)   +|αn−αn−1|(||ρU(xn−1)||+||μF(T(yn−1))||)  ≤(1−(ν−ρτ)αn)||xn−xn−1||   +M(1μ|rn−1−rn|+|βn−βn−1|+|αn−αn−1|),

where

(44) M=  max⁡{sup⁡n≥1(γ||A||σn+χn),     sup⁡n≥1(||Sxn−1||+||un−1||),     sup⁡n≥1(||ρU(xn−1)||+||μF(T(yn−1))||)}.

It follows from conditions (a)–(d) of Algorithm 11 and Lemma 9 that

(45) $$\begin{array}{c} \underset{n\to \infty }{\lim}||x_{n+1}-x_{n}||=0. \end{array}$$

Next, we show that lim⁡_*n*→*∞*_||*u*_*n*_ − *x*_*n*_|| = 0. Since x* ∈ Λ∩*F*(*T*) by using (34) and (37), we obtain

(46) ||xn+1−x∗||2  =〈PC[Vn]−x∗,xn+1−x∗〉  =〈PC[Vn]−Vn,PC[Vn]−x∗〉+〈Vn−x∗,xn+1−x∗〉  ≤〈αn(ρU(xn)−μF(x∗)+(I−αnμF)(T(yn)))    −(I−αnμF)(T(x∗)),xn+1−x∗〉  =〈αnρ(U(xn)−U(x∗)),xn+1−x∗〉   +αn〈ρU(x∗)−μF(x∗),xn+1−x∗〉   +〈(I−αnμF)(T(yn))     −(I−αnμF)(T(x∗)),xn+1−x∗〉  ≤αnρτ||xn−x∗||||xn+1−x∗||   +αn〈ρU(x∗)−μF(x∗),xn+1−x∗〉   +(1−αnν)||yn−x∗||||xn+1−x∗||  ≤αnρτ2(||xn−x∗||2+||xn+1−x∗||2)   +αn〈ρU(x∗)−μF(x∗),xn+1−x∗〉   +(1−αnν)2(||yn−x∗||2+||xn+1−x∗||2)  ≤(1−αn(ν−ρτ))2||xn+1−x∗||2   +αnρτ2||xn−x∗||2   +αn〈ρU(x∗)−μF(x∗),xn+1−x∗〉   +(1−αnν)2(βn||Sxn−x∗||2+(1−βn)||un−x∗||2)  ≤(1−αn(ν−ρτ))2||xn+1−x∗||2   +αnρτ2||xn−x∗||2   +αn〈ρU(x∗)−μF(x∗),xn+1−x∗〉   +(1−αnν)βn2||Sxn−x∗||2+(1−αnν)(1−βn)2   ×{||xn−x∗||2+γ(Lγ−1)||(TrnF2−I)Axn||2},

where the last inequality follows from (37), which implies that

(47) ||xn+1−x∗||2  ≤αnρτ1+αn(ν−ρτ)||xn−x∗||2   +2αn1+αn(ν−ρτ)〈ρU(x∗)−μF(x∗),xn+1−x∗〉   +(1−αnν)βn1+αn(ν−ρτ)||Sxn−x∗||2+(1−αnν)(1−βn)1+αn(ν−ρτ)   ×{||xn−x∗||2+γ(Lγ−1)||(TrnF2−I)Axn||2}  ≤αnρτ1+αn(ν−ρτ)||xn−x∗||2   +2αn1+αn(ν−ρτ)〈ρU(x∗)−μF(x∗),xn+1−x∗〉   +||xn−x∗||2+(1−αnν)βn1+αn(ν−ρτ)||Sxn−x∗||2   −(1−αnν)(1−βn)γ(1−Lγ)1+αn(ν−ρτ)||(TrnF2−I)Axn||2.

Then from the above inequality, we get

(48) (1−αnν)(1−βn)γ(1−Lγ)1+αn(ν−ρτ)||(TrnF2−I)Axn||2  ≤αnρτ1+αn(ν−ρτ)||xn−x∗||2+2αn1+αn(ν−ρτ)   ×〈ρU(x∗)−μF(x∗),xn+1−x∗〉   +βn||Sxn−x∗||2+||xn−x∗||2−||xn+1−x∗||2  ≤αnρτ1+αn(ν−ρτ)||xn−x∗||2+2αn1+αn(ν−ρτ)   ×〈ρU(x∗)−μF(x∗),xn+1−x∗〉   +βn||Sxn−x∗||2+(||xn−x∗||+||xn+1−x∗||)   ×||xn+1−xn||.

Since *γ*(1 − *Lγ*) > 0, lim⁡_*n*→*∞*_||*x*_*n*+1_ − *x*_*n*_|| = 0, *α*_*n*_ → 0, and *β*_*n*_ → 0, we obtain

(49) $$\begin{array}{c} \underset{n\to \infty }{\lim}||\left(T_{r_{n}}^{F_{2}}-I\right)Ax_{n}||=0. \end{array}$$

Since *T*_*r*_*n*__^*F*_1_^ is firmly nonexpansive, we have

(50) ||un−x∗||2  =||TrnF1(xn+γA∗(TrnF2−I)Axn)−TrnF1(x∗)||2  ≤〈un−x∗,xn+γA∗(TrnF2−I)Axn−x∗〉  =12{||un−x∗||2+||xn+γA∗(TrnF2−I)Axn−x∗||2     −||un−x∗−[xn+γA∗(TrnF2−I)Axn−x∗]||2}  =12{||un−x∗||2+||xn+γA∗(TrnF2−I)Axn−x∗||2     −||un−xn−γA∗(TrnF2−I)Axn||2}  ≤12{||un−x∗||2+||xn−x∗||2     −||un−xn−γA∗(TrnF2−I)Axn||2}  =12{||un−x∗||2+||xn−x∗||2     −[||un−xn||2+γ2||A∗(TrnF2−I)Axn||2       −2γ〈un−xn,A∗(TrnF2−I)Axn〉]},

where the last inequality follows from (34) and (37). Hence, we get

(51) ||un−x∗||2≤||xn−x∗||2−||un−xn||2 +2γ||Aun−Axn||||(TrnF2−I)Axn||.

From (46) and the above inequality, we have

(52) ||xn+1−x∗||2  ≤(1−αn(ν−ρτ))2||xn+1−x∗||2+αnρτ2||xn−x∗||2   +αn〈ρU(x∗)−μF(x∗),xn+1−x∗〉   +(1−αnν)2(βn||Sxn−x∗||2+(1−βn)||un−x∗||2)  ≤(1−αn(ν−ρτ))2||xn+1−x∗||2+αnρτ2||xn−x∗||2   +αn〈ρU(x∗)−μF(x∗),xn+1−x∗〉+(1−αnν)2   ×{βn||Sxn−x∗||2+(1−βn)     ×(||xn−x∗||2−||un−xn||2       +2γ||Aun−Axn||||(TrnF2−I)Axn||)},

which implies that

(53) ||xn+1−x∗||2 ≤αnρτ1+αn(ν−ρτ)||xn−x∗||2+2αn1+αn(ν−ρτ)  ×〈ρU(x∗)−μF(x∗),xn+1−x∗〉  +(1−αnν)βn1+αn(ν−ρτ)||Sxn−x∗||2+(1−αnν)(1−βn)1+αn(ν−ρτ)  ×{||xn−x∗||2−||un−xn||2    +2γ||Aun−Axn||||(TrnF2−I)Axn||} ≤αnρτ1+αn(ν−ρτ)||xn−x∗||2+2αn1+αn(ν−ρτ)  ×〈ρU(x∗)−μF(x∗),xn+1−x∗〉  +(1−αnν)βn1+αn(ν−ρτ)||Sxn−x∗||2  +||xn−x∗||2+(1−αnν)(1−βn)1+αn(ν−ρτ)  ×{−||un−xn||2+2γ||Aun−Axn||||(TrnF2−I)Axn||}.

Hence

(54) (1−αnν)(1−βn)1+αn(ν−ρτ)||un−xn||2 ≤αnρτ1+αn(ν−ρτ)||xn−x∗||2  +2αn1+αn(ν−ρτ)〈ρU(x∗)−μF(x∗),xn+1−x∗〉  +(1−αnν)βn1+αn(ν−ρτ)||Sxn−x∗||2  +2(1−αnν)(1−βn)γ1+αn(ν−ρτ)||Aun−Axn||||(TrnF2−I)Axn||  +||xn−x∗||2−||xn+1−x∗||2 ≤αnρτ1+αn(ν−ρτ)||xn−x∗||2  +2αn1+αn(ν−ρτ)〈ρU(x∗)−μF(x∗),xn+1−x∗〉  +(1−αnν)βn1+αn(ν−ρτ)||Sxn−x∗||2  +2(1−αnν)(1−βn)γ1+αn(ν−ρτ)||Aun−Axn||||(TrnF2−I)Axn||  +(||xn−x∗||+||xn+1−x∗||)||xn+1−xn||.

Since lim⁡_*n*→*∞*_||*x*_*n*+1_ − *x*_*n*_|| = 0, *α*_*n*_ → 0, *β*_*n*_ → 0, and lim⁡_*n*→*∞*_||(*T*_*r*_*n*__^*F*_2_^ − *I*)*Ax*_*n*_|| = 0, we obtain

(55) $$\begin{array}{c} \underset{n\to \infty }{\lim}||u_{n}-x_{n}||=0. \end{array}$$

Now, let *z* ∈ Λ∩*F*(*T*); since *T*(*x*_*n*_) ∈ *C*, we have

(56) ||xn−T(xn)|| ≤||xn−xn+1||+||xn+1−T(xn)|| =||xn−xn+1||+||PC[Vn]−PC[T(xn)]|| ≤||xn−xn+1||  +||αn(ρU(xn)−μF(T(yn))+T(yn)−T(xn))|| ≤||xn−xn+1||  +αn||ρU(xn)−μF(T(yn))||+||yn−xn|| ≤||xn−xn+1||+αn||ρU(xn)−μF(T(yn))||  +||βnSxn+(1−βn)un−xn|| ≤||xn−xn+1||+αn||ρU(xn)−μF(T(yn))||  +βn||Sxn−xn||+(1−βn)||un−xn||.

Since lim⁡_*n*→*∞*_||*x*_*n*+1_ − *x*_*n*_|| = 0,  *α*_*n*_ → 0,  *β*_*n*_ → 0,  ||*ρU*(*x*_*n*_) − *μF*(*T*(*y*_*n*_))||, and ||*Sx*_*n*_ − *x*_*n*_|| are bounded and lim⁡_*n*→*∞*_||*x*_*n*_ − *u*_*n*_|| = 0, we obtain

(57) $$\begin{array}{c} \underset{n\to \infty }{\lim}||x_{n}-T\left(x_{n}\right)||=0. \end{array}$$

Since {*x*_*n*_} is bounded, without loss of generality, we can assume that *x*_*n*_⇀x* ∈ *C*. It follows from Lemma 6 that x* ∈ *F*(*T*). Therefore *w*_*w*_(*x*_*n*_) ⊂ *F*(*T*).

**Theorem 15**

The sequence {*x*_*n*_} generated by Algorithm 11 converges strongly to *z*, which is the unique solution of the variational inequality

(58) $$\begin{array}{c} \langle \rho U\left(z\right)-\mu F\left(z\right),x-z\rangle \leq 0,\;\forall x\in \Lambda \cap F\left(T\right). \end{array}$$

**Proof**

Since {*x*_*n*_} is bounded *x*_*n*_⇀*w* and from Lemma 14, we have *w* ∈ *F*(*T*). Next, we show that *w* ∈ *EP*⁡(*F*_1_). Since *u*_*n*_ = *T*_*r*_*n*__^*F*_1_^(*x*_*n*_ + γA*(*T*_*r*_*n*__^*F*_2_^ − *I*)*Ax*_*n*_), we have

(59) F1(un,y)+1rn〈y−un,un−xn〉  −1rn〈y−un,γA∗(TrnF2−I)Axn〉≥0, ∀y∈C.

It follows from monotonicity of *F*_1_ that

(60) −1rn〈y−un,γA∗(TrnF2−I)Axn〉  +1rn〈y−un,un−xn〉≥F1(y,un), ∀y∈C,

(61) −1rnk〈y−unk,γA∗(TrnkF2−I)Axnk〉  +〈y−unk,unk−xnkrnk〉≥F1(y,unk), ∀y∈C.

Since lim⁡_*n*→*∞*_||*u*_*n*_ − *x*_*n*_|| = 0, lim⁡_*n*→*∞*_||(*T*_*r*_*n*__^*F*_2_^ − *I*)*Ax*_*n*_|| = 0, and *x*_*n*_⇀*w*, it easy to observe that *u*_*n*_*k*__ → *w*. It follows by Assumption 3(iv) that *F*_1_(*y*, *w*) ≤ 0, for all *y* ∈ *C*.

For any 0 < *t* ≤ 1 and *y* ∈ *C*, let *y*_*t*_ = *ty* + (1 − *t*)*w*; we have *y*_*t*_ ∈ *C*. Then, from Assumptions 3((i) and (iv)), we have

(62) 0=F1(yt,yt)≤tF1(yt,y)+(1−t)F1(yt,w)≤tF1(yt,y).

Therefore *F*_1_(*y*_*t*_, *y*) ≥ 0. From Assumption 3(iii), we have *F*_1_(*w*, *y*) ≥ 0, which implies that *w* ∈ *EP*⁡(*F*_1_).

Next, we show that *Aw* ∈ *EP*⁡(*F*_2_). Since {*x*_*n*_} is bounded and *x*_*n*_⇀*w*, there exists a subsequence {*x*_*n*_*k*__} of  {*x*_*n*_} such that *x*_*n*_*k*__ → *w* and since *A* is a bounded linear operator, *Ax*_*n*_*k*__ → *Aw*. Now set *v*_*n*_*k*__ = *Ax*_*n*_*k*__ − *T*_*r*_*n*_*k*___^*F*_2_^ *Ax*_*n*_*k*__. It follows from (49) that lim⁡_*k*→*∞*_ *v*_*n*_*k*__ = 0 and *Ax*_*n*_*k*__ − *v*_*n*_*k*__ = *T*_*r*_*n*_*k*___^*F*_2_^ *Ax*_*n*_*k*__. Therefore from the definition of *T*_*r*_*n*_*k*___^*F*_2_^, we have

(63) F2(Axnk−vnk,y)  +1rnk〈y−(Axnk−vnk),     (Axnk−vnk)−Axnk〉≥0, ∀y∈C.  

Since *F*_2_ is upper semicontinuous in first argument, taking lim sup to above inequality as *k* → *∞* and using Assumption 3(iv), we obtain

(64) $$\begin{array}{c} F_{2}\left(Aw,y\right)\geq 0\;\forall y\in C, \end{array}$$

which implies that *Aw* ∈ *EP*⁡(*F*_2_) and hence *w* ∈ Λ.

Thus we have

(65) $$\begin{array}{c} w\in \Lambda \cap F\left(T\right). \end{array}$$

Observe that the constants satisfy 0 ≤ *ρτ* < *ν* and

(66) k≥η ⟺k2≥η2 ⟺1−2μη+μ2k2≥1−2μη+μ2η2 ⟺1−μ(2η−μk2)≥1−μη ⟺μη≥1−1−μ(2η−μk2) ⟺μη≥ν.

Therefore from Lemma 7, the operator *μF* − *ρU* is *μη* − *ρτ* strongly monotone, and we get the uniqueness of the solution of the variational inequality (58) and denote it by *z* ∈ Λ  ∩  *F*(*T*).

Next, we claim that lim⁡sup⁡_*n*→*∞*_〈*ρU*(*z*) − *μF*(*z*), *x*_*n*_ − *z*〉≤0. Since {*x*_*n*_} is bounded, there exists a subsequence {*x*_*n*_*k*__} of {*x*_*n*_} such that

(67) limsup⁡n→∞〈ρU(z)−μF(z),xn−z〉 =limsup⁡k→∞〈ρU(z)−μF(z),xnk−z〉 =〈ρU(z)−μF(z),w−z〉≤0.

Next, we show that *x*_*n*_ → *z*. Consider

(68) ||xn+1−z||2 =〈PC[Vn]−z,xn+1−z〉 =〈PC[Vn]−Vn,PC[Vn]−z〉+〈Vn−z,xn+1−z〉 ≤〈αn(ρU(xn)−μF(z))+(I−αnμF)(T(yn))   −(I−αnμF)(T(z)),xn+1−z〉 ≤〈αnρ(U(xn)−U(z)),xn+1−z〉  +αn〈ρU(z)−μF(z),xn+1−z〉  +〈(I−αnμF)(T(yn))−(I−αnμF)(T(z)),xn+1−z〉 ≤αnρτ||xn−z||||xn+1−z||  +αn〈ρU(z)−μF(z),xn+1−z〉  +(1−αnν)||yn−z||||xn+1−z|| ≤αnρτ||xn−z||||xn+1−z||  +αn〈ρU(z)−μF(z),xn+1−z〉  +(1−αnν){βn||Sxn−Sz||+βn||Sz−z||        +(1−βn)||un−z||}||xn+1−z|| ≤αnρτ||xn−z||||xn+1−z||  +αn〈ρU(z)−μF(z),xn+1−z〉  +(1−αnν){βn||xn−z||+βn||Sz−z||        +(1−βn)||xn−z||}||xn+1−z|| =(1−αn(ν−ρτ))||xn−z||||xn+1−z||  +αn〈ρU(z)−μF(z),xn+1−z〉  +(1−αnν)βn||Sz−z||||xn+1−z|| ≤1−αn(ν−ρτ)2(||xn−z||2+||xn+1−z||2)  +αn〈ρU(z)−μF(z),xn+1−z〉  +(1−αnν)βn||Sz−z||||xn+1−z||

which implies that

(69) ||xn+1−z||2 ≤1−αn(ν−ρτ)1+αn(ν−ρτ)||xn−z||2  +2αn1+αn(ν−ρτ)〈ρU(xn)−μF(z),xn+1−z〉  +2(1−αnν)βn1+αn(ν−ρτ)||Sz−z||||xn+1−z|| ≤(1−αn(ν−ρτ))||xn−z||2+2αn(ν−ρτ)1+αn(ν−ρτ)  ×{1ν−ρτ〈ρU(z)−μF(z),xn+1−z〉    +(1−αnν)βnαn(ν−ρτ)||Sz−z||||xn+1−z||}.

Let *γ*_*n*_ = *α*_*n*_(*ν* − *ρτ*) and *δ*_*n*_ = (2*α*_*n*_(*ν* − *ρτ*)/(1 + *α*_*n*_(*ν* − *ρτ*))){(1/(*ν* − *ρτ*))〈*ρU*(*z*) − *μF*(*z*), *x*_*n*+1_ − *z*〉 + ((1 − *α*_*n*_ *ν*)*β*_*n*_/*α*_*n*_(*ν* − *ρτ*))||*Sz* − *z*||||*x*_*n*+1_ − *z*||}.

Since

(70) ∑n=1∞αn=∞,limsup⁡n→∞{1ν−ρτ〈ρU(z)−μF(z),xn+1−z〉    +(1−αnν)βnαn(ν−ρτ)||Sz−z||||xn+1−z||}≤0.

It follows that

(71) $$\begin{array}{c} \;\;\overset{\infty }{\underset{n=1}{\sum }}\gamma_{n}=\infty ,\;\;\underset{n\to \infty }{\lim\;\sup}\frac{\delta_{n}}{\gamma_{n}}\leq 0. \end{array}$$

Thus all the conditions of Lemma 9 are satisfied. Hence we deduce that *x*_*n*_ → *z*. This completes the proof.

**Remark 16**

In hierarchical fixed point problem (17), if *S* = *I* − (*ρU* − *μF*), then we can get the variational inequality (58). In (58), if *U* = 0, then we get the variational inequality 〈*F*(*z*), *x* − *z*〉 ≥ 0, for all *x* ∈ Λ∩*F*(*T*), which is just the variational inequality studied by Suzuki [27] extending the common set of solutions of a system of variational inequalities, a split equilibrium problem, and a hierarchical fixed point problem.

## 4. Conclusions

In this paper, we suggest and analyze an iterative method for finding the approximate element of the common set of solutions of (15)-(16) and (17) in real Hilbert space, which can be viewed as a refinement and improvement of some existing methods for solving a split equilibrium problem and a hierarchical fixed point problem. Some existing methods (e.g., [13, 14, 17–19, 21–23]) can be viewed as special cases of Algorithm 11. Therefore, the new algorithm is expected to be widely applicable.
